# Editorial

**Published:** 1856-01

**Authors:** 


### Editorial.

### A NEW YEAR'S GIFT.

"A thing of beauty" and of grace,
A dimple chin and smiling face,
Gems of art in porcelain ware,
These teeth are beauties I declare.

These expressions were forced from us by opening a small package addressed to us, and which came opportunely to hand at the close of last year. Whether designed or not we shall regard it as a gift for the New year, and hail it as an auspicious *omen* for the year 1856.

It is truly gratifying to see continued and indefatigable efforts put forth by the manufacturers of porcelain teeth to meet all the wants of the

Profession. Messrs. Jones, White & McCurdy appear determined to maintain their high position in this respect.

Celebrated painters are said to sketch every muscle, gesture and expression from living reality—selecting for their *studio* the fairest models which nature supplies. The mastery of art is to picture nature just as she is.

We have never been in the *studio* of Messrs. J. W. & McC., but from the specimens of their art now before us we must believe that some fair nymph has consented to reveal the hidden beauty of her mouth and set for at least one hour with her *ruby* lips apart, exposing for the benefit of mankind her roseate gums and beautiful dental gems. One or two specimens now before us are demonstrable evidence to us that at least one of these gentlemen have of late been worshippers at the shrine of beauty. Here we have the four central Incisors in two blocks, central and lateral together, like man and wife, the central a little the tallest and of course the largest standing easy and gracefully with conscious pride, feeling that he has beside him the delicate, symmetrical and lovely partner of life, who is softly leaning against him for support with her head gently touching his shoulder. The blood appears scarcely hid under the delicate mucous membrane of the gum. The body of the teeth appear as dense and firm as porcelain can be made, and the delicate shades of the teeth are mingled in life-like reality.

But we have another specimen which looks the man all over, and yet we think the most perfect of the kind we have ever seen. It is an upper denture of eight teeth in four blocks, here is the same life-like appearance of the gums—with an almost faint outline of the roots of the teeth seen beneath them. The roots of the cuspid teeth bulge up in perfect mockery of nature where we have a marked and exposed character. The whole denture is complete in every characteristic. The teeth round and short with smooth enamel, the organs look strong and compact, the shading of the teeth is of that yellow tint characteristic of an innatily good constitution, and the gums show that the constitutional vigor is not by any means as yet impaired. The healthful laws of nature have not been violated—art has made no blunder here and if the teeth themselves are not really beautiful, the combination makes them so. We cannot but think what a blunder it would be to put such teeth in a delicate lady's mouth, and such teeth are scarcely ever lost except by accident.

A specimen of a set of teeth for continuous gum work are as if moulded from nature, roots and all; only the point of the roots nipped off—yet we cannot but think a mistake was made by closing up the dental foramen. These teeth with various others of different style and all essential to meet the varied wants of the profession, are now being regularly supplied to their agent in this City, Dr. J. M. Brown, whose reputation as a connoisseur in supplying the profession with needful articles is too well known to need commendation from us.

MISSISSIPPI VALLEY ASSOCIATION OF DENTAL SURGEONS.

We call special attention to the following communication of the .Executive Committee of the Mississippi Valley Association of Dental Surgeons, and hope that the members of the society and those in the profession who may wish to take part in the discussion on these subjects will come prepared to make such discussions profitable and interesting. We shall propose a regular reporter to take down for publication the remarks made by every member. It will be seen that every question proposed by the committee is important, and must draw out much important practical knowledge.

The 9th and 10th questions embrace a fine field for discussion and if entered into aright will elicit much useful instruction.

We hope it will be recollected that this society convenes on Wednesday 20th February next, at 10 o'clock A. M. in the Lecture room of the Ohio College of Dental Surgery.

MR. EDITOR,

The Executive Committee of the Mississippi Valley Association of Dental Surgeons, thinking that it would be well for the members to have some time to consider upon the subjects brought up for discussion before the Association, will present in the order of business—the following questions, and hope that not only the members but all others interested will come fully prepared to discuss them.

1st. Does continuous gum work bear the test of experience?

2d. What is the best method of preparing gold for filling teeth?

3d. What is the best method of drying cavities previous to filling, also partially filled cavities, when accidentally wet with saliva before the work is completed?

4th. Has block work any advantages over single teeth, that are not counterbalanbed by corresponding disadvantages—if it has, what are they?

5th. What are the best methods of, and the best instruments for separating teeth preparatory to filling?

6th. How can the natural teeth that require filling be most effectually preserved?

7th. Is Gutta Percha reliable as a base for artificial teeth, if so, the best method of applying it?

8th. Can artificial teeth when moulded on porcelain base be worn with advantage in any case?

9th. What are the various pathological conditions of inflamed dentine, and what circumstances modify its treatment?

10th. What is the modus operandi of arsenic, Chl. of zinc, Nit. of silver, creosote, tannin, etc. when applied to inflamed dentine?

H. R. SMITH,<br>

J. DOUTHETT, } Committee.<br>

J. P. ULREY.

---

OHIO DENTAL COLLEGE ASSOCIATION.

We would remind the Stockholders of the College, and members of this Association, that the next annual meeting of the College Association takes place on the third Monday (18th) of February next, at 2 o'clock P. M. Business of importance will be brought before the Association, and it is important that every stockholder and member should be present. Members of the profession generally are invited to attend. The commencement exercises of the College will take place on Wednesday evening, the 20th of February.

We hope the examining committee for the graduating class will be on hand as early on Monday forenoon as possible.

---

SINGULAR DENTAL SPECIMEN.

The postscript of a recent letter from Dr. E. P. Peters, of Wabash, Ind., gives us the following description of a two fanged inferior lateral incisor, which we hope to place in the College Museum soon. We do not now recollect of ever having seen such a case. The postscript is as follows:

P. S. A short time ago I extracted, from the mouth of a lady, an *inferior lateral incisor*, having two separate and distinct fangs. I mention it because it is something entirely new to me, though you may have seen the same thing yourself. If you have not, and have any desire to see it, by informing me through the Register or by letter, I will forward it to you.

Yours, etc. E. P. P.

Dr. J. TAYLOR.

---

FOX AND HARRIS.

This work which is just issued from the press of Lindsay & Blackiston, is a fair sample of the chaste and elegant style which they maintain in their numerous publications.

There is no English work which deserves to be more constantly kept in the hands of the profession than Fox's, not indeed that the practice which he gives us in all its detail is adapted to the present state of American Dentistry, but the student can justly view it as one of the foundation stones (and that one of the broadest) on which our profession now rests. In some things Fox has been but little improved—take for instance the treatment of irregularity. This work is as free from superfluous matter as any work in the profession.

Prof. Harris has by his numerous additions completely modernized it, or if we may use the term—Americanized it, and in doing this has made it a far more valuable work for the student of dentistry.

The work contains two hundred and fifty illustrations, some of these are exceedingly fine and are of great value to the student, take for example plates three, four, six and seven, and they exhibit the different condition of the teeth during their germination and eruption, and if carefully studied will enable us to see how very pernicious may become the interference of art in the eruption of the permanent organs.

---

THE MEDICAL COUNSELLOR AND WEEKLY GAZETTE OF THE MEDICAL AND PHYSICAL SCIENCES.

We have now on our table the Nov. No. of this new weekly periodical, R. Hills M. D. Editor and Proprietor, Columbus, O. Among the articles of interest the present number contains "Observations at the Bedside." Treatment of sciatica with chloroform. Complete dislocation of the lower jaw reduced by a new method. Antidote to stychnine. A case of Rupture of the uterus and recovery. The work is gotten up in neat style and we should think would be well sustained by the profession.

---

DISCOVERY OF THE CAUSE, NATURE, CURE AND PREVENTION OF EPIDEMIC CHOLERA. BY M. L. KNAPP, M. D., ETC.

Dr Knapp proposes publishing by subscription four Essays explanatory of his views of the nature, cause and cure of cholera. The present specimen number containing about fifty pages, is the first of the quarterly series which is to make when complete a volume of about 500 pages, at $4 00 or $3 00 if paid in advance.

Dr. Knapp takes the ground that cholera is a scorbutic disease and on this head remarks that "I am forced to the conclusion, that cholera is but a modified form of scorbutus, or a younger sister-scourge of the same parentage; probably better expressed by calling it a hemorrhagic termination, or a manifestation of the dying phenomena of scorbutus," many plausible arguments and facts are brought to support this position, but as yet we have not seen enough to convince—the Dr. is a fine writer, and the work will be worth careful study.

---

THE MEDICAL SPECIALIST AND JOURNAL OF DISEASES OF THE CHEST.

We have received the first and second numbers of this monthly by Robert Hunter M. D., 828 Broadway N. Y. These numbers are principally taken up with the character of the diseases of the chest, and the principles of inhalation in their treatment. We hope to see in future numbers the more practical applicability of this practice discussed with the particular remedies used for special cases

The Dr. writes with a ready pen, and the work is gotten up with considerable taste.

We are decidedly in favor of specialities in medical science, believing that the interest of the profession and humanity can best be promoted by making special diseases a particular study.

We devote our time and talents, so far as we have any, to a speciality and feel that we have as much on our hands as we can well attend to. We cheerfully put the Specialist on our exchange list. The Specialist is published at $1 00 per year in advance.

---

BISULPHURET OF CARBON.

This is used by by M. Ellepsen as a solvent for gutta percha—perhaps the profession may turn it to some account in preparing this article for dental purposes.

### THE PHILADELPHIA MEDICAL AND SURGICAL JOURNAL. EDITED BY JAMES BRYAN, A. M., M. D.

This monthly periodical is sustained with mueh ability and has now reached the seventh number of Vol. IV. We have as yet received but two or three numbers, bnt have been much interested in their contents. The Journal is issued at $1 50 per year in advance.

---

### FRANK LESLIE'S GAZETTE OF FASHION, AND FRANK LESLIE'S NEW-YORK JOURNAL.

We have received the specimen numbers and prospectus of these works. They will be handsomely illustrated, and we can at least with much propriety commend the Gazette of Fashion to the profession. It would make awelcome to many ladies on the center table of the reception room. That is, provided our own wives allowed it to remain there.

That the work will be neatly gotten up and meet the wants of the ladies there can be no doubt.

---

### PRONOUNCING MEDICAL LEXICON.

This is a new and very convenient book for the medical student and indeed from its compactness and brevity exceedingly useful to the physician. All the words are first given in their ordinary orthography and then is added the pronunciation in phonetic characters. We like its conciseness and think it will meet with extensive sale. It is the work of Prof. C. H. Cleaveland, M. D., Cincinnati, Ohio.

---

### DR. B. A. SATTERTHWAITE'S IMPROVEMENT.

We noticed some time since the efforts that were being made by Drs. Satterthwaite and Loomis to get out entire upper and lower dentures with porcelain. That is a continuous gum, teeth and base all united together without any metal whatever. We had our doubts about the possibility of *baking* such an operation and not so changing it as to prevent any fit at all. A month or two since we sent to Dr. Satterthwaite a plaster model from which a suction plate had been obtained, with simply the bite of the uuder teeth. In a few days the Dr. returned us a set of teeth most beautifully gotten up and which fit exceedingly well to the mouth—the porcelain base which covers the palate is quite thin and yet this and the body around the teeth is all covered with a beautiful and life like gum. The whole operation is quite light and if it had the strength of gold work we should think it at near perfection as we expect to get.

The Dr. uses, he informs us, this kind of work very successfully in his practice and his patients are pleased with it.

---

### A. J. WATTS' IMPROVED PREPARED GOLD.

We have been repeatedly assured by different members of the profession that this article as now prepared is of superior quality, appearing to combine all the qualities so much needed in gold for filling teeth. We are satisfied that prepared or crystal gold meets some exigencies for filling certain cavities, which makes it a valuable acquisition to the profession—our experience with these forms of gold has not been as yet very extensive, yet sufficiently so to warrant the belief that good plugs can be made with it. We have however not found the labor diminished by its use, and for cavities of difficult access, and also such as are difficult to be kept dry, we think it will hardly supersede good foil. We have been so long using blocks (or cylinders) in plugging that we are somewhat wedded perhaps to that mode. Yet we shall test as we think thoroughly this improved preparation of gold. It is for sale by Dr. J. M. Brown, Dental Depot, Cincinnati, Ohio.

ERRATA.

In looking over the last number of the Register we have been mortified to find an error in spelling the name of our friend Dr. A. S. Talbert, of Lexington Ky. It occurs in an article headed "Dental Obturator." The T. is left off his name or placed as one of the initials. We corrected this in the proof, but by some oversight it went through the press without correction.

OBITUARY.

We are pained to hear of the recent decease of Dr. T. S. Warring, of Zanesville, Ohio, after a somewhat protracted illness. The Dr. had been for many years in the profession, and had acquired by persevreing industry an enviable reputation. For the last year or more he has had associated with him in practice Dr. Streeper, who we are assured, shares largely the confidence of the public.

